# Quality and safety issues related to traditional animal medicine: role of taurine

**DOI:** 10.1186/1423-0127-17-S1-S44

**Published:** 2010-08-24

**Authors:** Kyoko Takahashi, Yuko Azuma, Kayoko Shimada, Tadashi Saito, Masaya Kawase, Stephen W Schaffer

**Affiliations:** 1 The Museum of Osaka University, Toyonaka, Osaka, Japan; 2 Graduate School of Pharmaceutical Sciences, Osaka University, Suita, Osaka, Japan; 3 Radioisotope Research Center, Osaka University, Suita, Osaka, Japan; 4 Nagahama Institute of Bio-Science and Technology, Nagakama, Shiga, Japan; 5 School of Medicine, University of South Alabama, Mobile, AL, USA

## Abstract

**Background:**

*Calculus Bovis* (:*C.Bovis*) is one of the most precious and commonly-used medicinal materials in Japan and China. As the natural occurrence is very rare, a source of supply for *C. Bovis* is far behind the actual need and great efforts have been taken for some substitutes of natural *C. Bovis*. Unfortunately, very little information is available on the quality and/or clinical efficacy of medication based on *C. Bovis*. To ensure sustainable use of traditional therapeutic agents derived from *C. Bovis*, we felt that several issues needed to be addressed: 1) the source of the *C. Bovis* materials and quality control; 2) the role of taurine in the efficacy of *C. Bovis*.

**Methods:**

Nine samples of natural *C. Bovis* and its substitutes were collected. ICP-MS was used for elemental analysis and the characterization was performed by principal component analysis (PCA) and soft independent modeling of class analogy (SIMCA) as multivariate approaches. The efficacy of *C. Bovis* was evaluated for morphology, viability and beating pattern on cultured cardiac myocytes and/or fibroblasts.

**Results:**

PCA and multi-elemental focus was effective in discriminating *C. Bovis* samples derived from different habitats. A satisfactory classification using SIMCA was obtained among Australia *C. Bovis*, other habitats and the substitutes. Australian samples had better batch uniformity than other habitats and were composed of fewer elements. We have used Australian* C. Bovis* for assessment on its bioactive compounds. Rat cardiac cells incubated with *C. Bovis* extract (0.01-0.1mg/ml) maintained normal morphology, viability and beating pattern. Cardiac myocytes and fibroblasts treated for 48 h with CA (0.5mM) or DCA (0.1mM) caused cell injury, as reflected by changes in appearance and a reduction of viability detected by the MTS assay. In cardiomyocytes, 0.5 h exposure of CA (0.5mM) markedly decreased the velocity ratio of beating, whereas the simultaneous addition of 1 mM taurine largely prevented the decrease.

**Conclusions:**

The multi-elemental focus provided some references for the quality control and the efficacy of *C. Bovis*. Taurine partly attenuated the harmful actions of bile acids. It is plausible that the relationship between taurine and the bile acids contributes to therapeutic effect of *C. Bovis*.

## Background

*Calculus Bovis* (Goo in Japanese, Niuhuang in Chinese, the gallstone of *Bos Taurus domesticus* Gmmelin) is one of the most precious and commonly-used medicinal materials in Japan and China. Its use was first recorded in “Shennong Bencao Jing” (Divine Farmer’s Materia Medica Classic) more than two thousand years ago, and now it has been used in 650 out of the 4500 traditional Chinese medicines [1,2]. In the 230 cardioactive types of Japanese OTC drugs, 228 drugs contain *C. Bovis,* which has the effects of sedation, anti-hyperspasmia, relieving fever, diminishing inflammation and normalizing function of the gallbladder [3,4].  A source of supply for *C. Bovis* is far behind the actual need since the natural occurrence of *C. Bovis* is very rare. In China, great efforts have been taken for some substitutes of natural *C. Bovis*. The safety and efficacy of *C. Bovis* is closely correlated with quality of the source materials. Unfortunately, very little information is available on the quality and/or clinical efficacy of medication based on traditional animal sources.

We have previously reported on the anti-arrhythmic actions of *C. Bovis* as a traditional knowledge-product and suggested these effects are partly mediated by taurine [3]. To ensure sustainable use of traditional therapeutic agents derived from *C. Bovis*, we felt that several issues needed to be addressed: 1) the source of the *C. Bovis* preparation and quality control; 2) the role of taurine in the cardio-active efficacy of *C. Bovis*.

## Materials and methods

### Samples and reagents

Thirteen samples of natural *C. Bovis* and its substitutes (artificial *C. Bovis,* cultured *C. Bovis*) from various places were collected and are listed in Table 1. Nine batches of natural* C. Bovis* samples produced from Australia, Argentina, Brazil, Guatemala, Mexico and Kenya were purchased from Tochimoto Tenkai-Do (Osaka, Japan). All materials used in this study were stored in the Department of Applied Pharmacognosy, the Museum of Osaka University, Japan. Cholic acid (CA), deoxycholic acid (DCA) and glycine-/taurine-conjugated bile acids were purchased from Nacalai Tesque, Inc. (Kyoto, Japan). Other regents were of analytical grade.

**Table 1:** Summary of investigated samples

No.	Species	Properties	Source	Collection site	Collection date
1	Natural C.bovis	Natural, PW ^1)^	Tochimoto co.	Australia-1	2006
2	Natural C.bovis	Natural, PW	Tochimoto/Toyama	Australia-2	2001
3	Natural C.bovis	Natural, PW	Tochimoto/Toyama	Argentine	2001
4	Natural C.bovis	Natural, PW	Tochimoto/Toyama	Brazil	2001
5	Natural C.bovis	Natural, PW	Tochimoto/Toyama	Guatemala	2001
6	Natural C.bovis	Natural, PW	Tochimoto/Toyama	Mexico	2001
7	Natural C.bovis	Natural, PW	Tochimoto/Toyama	Kenya	2001
8	Natural C.bovis	Natural, CL^2)^	Takahashi K . ^3)^	China	2007
9	Natural C.bovis	Natural, CL	Osaka univ. ^4)^	India	1978

10	Artificial C.bovis	In vitro prepared, PW	Takahashi K .	China	2007
11	Artificial C.bovis	In vitro prepared, PW	Osaka univ.	China	1971
12	Artificial cultured C.bovis	In vitro cultured, CL	Tochimoto co.	China, Hubei	2009
13	Artificial cultured C.bovis	In vitro cultured, CL	Tochimoto co.	China, Anhui	2009

The abbreviations of properties: ^1)^ PW: powder, ^2)^CL: clod. ^3)^ Samples were purchased from local stores in Shenyang by Takahashi K. ^4)^ Historical specimens stored in the museum of Osaka University.

### Preparation of C*alculus. Bovis*-extract and taurine measurement

Water or DMSO was used as the solvent to extract the desired components. Natural *C. Bovis* contains a high concentration of bile acid, which adversely affects cells by disrupting their cell membrane. Each concentration of major bile acids in *C. Bovis* (100% DMSO) was equivalent to 0.5 mM CA, 0.1 mM DCA and 0.5 mM taurocholic acid (:TCA), respectively [3]. To minimize the extraction of these bile acids, water or DMSO/water (=2:1) was used as the solvent to extract the desired components. Ten mg of well-pulverized crude drug was extracted with 5 ml of water at room temperature. The extract was centrifuged at 12000 x g for 20 min at 4°C. A final concentration of *C.Bovis* extract to the culture medium was 0.01-0.1mg/ml according to the human OTC doses.

The taurine or bile acids content of *C. Bovis* derived samples was measured by the procedure using ultra performance liquid chromatography (LaChromUltra, HITACHI, Inc., Tokyo, Japan) equipped with a L-2485U fluorescence detector or L-2400U UV detector , respectively.

### ICP-MS measurement and multivariate analysis

Major and trace elements in materials were measured by using inductively coupled plasma mass spectrometry (:ICP-MS) [5]. The *C. bovis* powder (10 mg) was added to 1ml of HNO_3_ (Nacalai Tesque, Kyoto, Japan), vortexed and left standing overnight at room temperature. Then, 100μl of samples were diluted with 9.9 ml of water and filtered through 0.45 μm pore size hydrophilic PTFE membrane filter (Millipore, Billerica, MA). ICP-MS analysis was performed on the Agilent 7500 Series ICP-MS (Agilent Technologies, Inc., Santa Clara, CA). Among the attained data, the elements which were detected as more than 1μg/l in the solvent were selected. Each data in the chart was shown as relative concentration. Principal component analysis (PCA) and soft independent modeling of class analogy (SIMCA) were used as exploratory techniques and classification procedures, respectively [6]. Variables such as Na, Mg, Al, K, Ca, V, Cr, Mn, Fe, Co, Ni, Cu, Zn, Ge, As, Mo, Hg and Pb have been used as discriminating factors.

### Cell culture and assessment on bioactive compounds of *Calculus Bovis*

Primary cultures of cardiac cells from 1-day-old Wister rats were prepared according to the methods described previously [7]. All experimental procedures were approved by the Animal Care Committee of Osaka University and conformed to international guidelines.

Cardiac cells were seeded in a 96-well plate and were determined by MTS assay using CellTiter 96® aqueous one solution reagent (Promega Corporation) [8]. After 48 h incubation in serum-containing culture medium, medium was transferred to serum-free medium. After 24h incubation, cells were treated with *C. Bovis*, bile acids and taurine for 48h. Following removal of the *C. Bovis*, cells were washed with phosphate-buffered saline (PBS) twice. The cells were then incubated in serum free maintenance medium (100μl) with one solution reagent (20μl) for 3h. Cell viability was defined as the ratio (expressed as a percentage) of absorbance of treated cells to untreated cells at 490nm.

The beating status of cultured myocardial cells was monitored with an inverted phase-contrast microscope and was measured by using a photosenser equipped with a microcomputer (P-200, Adachi Co.,Osaka). The shape and location of each cell in the dish were recorded before initiating the experiments [9]. The beating properties of the same cells were monitored following the chosen perturbation.

### Statistic analysis

Statistical significance was determined by the Student's t-test. Each value was expressed as the mean ± S.E. Differences were considered statistically significant when the calculated P value was less than 0.05.

## Results

### Quality characterization of *Calculus Bovis* using pattern recognition techniques and multiple element data

It is an important issue to comprehensively evaluate the different species of *C. Bovis*, so as to ensure the clinical efficacy of this medicinal material. The provisions of Chinese Pharmacopeia [4] for some substitutes of natural *C. Bovis* were summarized in Table 2. One has been so-called artificial *C. Bovis,* being a mixture of bile salts, bilirubin, taurine and some other ingredients that have been found and believed to have contributed to the therapeutic effects of natural *C. Bovis*. Another is the so-called cultured *C. Bovis,* which is either the induced gallstones in animals in vivo, or those produced in vitro under the conditions mimicking the gallstone formation process in vivo [2].

**Table 2:** Charcteristics of *Calculus Bovis* in Chinese Pharmacopoeia 2005

Crude drugs	Ingredients	Compounds
Natural Calculus Bovis, Niuhuang CALCULUS BOVIS	*Bos taurus domesticus* Gmelin	CA : ≥ 4.0% Bilirubin ≥ 35.0% Total ash ≤ 10.0%

Artificial Calculus Bovis Rengong Niuhuang CALCULUS BOVIS ARTIFACTUS	Ox bile-powder (CA: ≥ 42.0%) Cholic acid:CA ( ≥ 80.0%) Hyodeoxycholic acid:HCA Taurine Bilirubin ( ≥ 90%) Cholesterol, Trace elements	CA : ≥ 13.0% Bilirubin : ≥ 0.63%

In-vitro cultured Calculus Bovis Tiwai Peiyu Niuhuang CALCULUS BOVIS SATIVUS	Fresh bile of *Bos taurus* *domesticus* Gmelin Cholic acid:CA ( ≥ 95.0%) Ca Bilirubinacet complexes ( CA ≥ 43.0 %) Deoxycholic acid:DCA ( ≥95.0 %)	CA : ≥ 6.0% Bilirubin : ≥ 35.0%

China Pharmacopoeia Committee: *Pharmacopoeia of the People’s Republic of China, 1^st^ Div (2005 edition)* Beijing, China Chemical Industry Press 2005

On the other hand, reference values for *C. Bovis* in Japanese Pharmacopeia [10] is as follows: spherical or massive stone, 1-4 cm in diameter; externally yellow-brown to red-brown; light, fragile and easily broken. Fractured surface shows yellow-brown to red-brown annular rings, often containing white granular substances or thin layers in these annular rings. Odor, slight; taste, slightly bitter, followed by slight sweetness. Namely, the form, colour, smell and very importantly the taste are some of the sensory indicators, on the basis of traditional knowledge, to test for quality.

**Figure 1:**
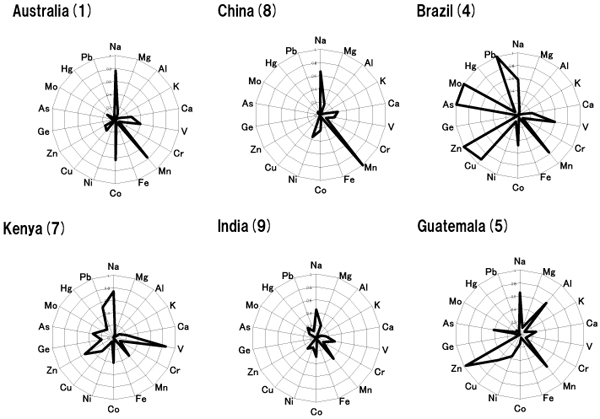
**Multiple element patterns of natural *Calculus Bovis* samples derived from different habitats** The number in parentheses matched to the number of samples in Table 1. The elements which were detected more than 1 ng/l in the solvent were selected. Each data in the chart was shown as relative concentration. Relative concentration was calculated by setting the maximum concentration of each element contained in samples as 1 (n=3).

Therefore, mulitiple elements in *C. Bovis* materials of natural and substitutes were determined by using ICP-MS. Eighteen element patterns for each sample of *C. Bovis* were illustrated in Figure 1. High differences can be observed in the elemental composition among the samples, especially for some elements such as Zn and Al. Na and Mn are similar components in the samples of natural *C. Bovis*. Australian samples were composed of fewer elements without toxic metals such as As and Hg.

**Figure 2:**
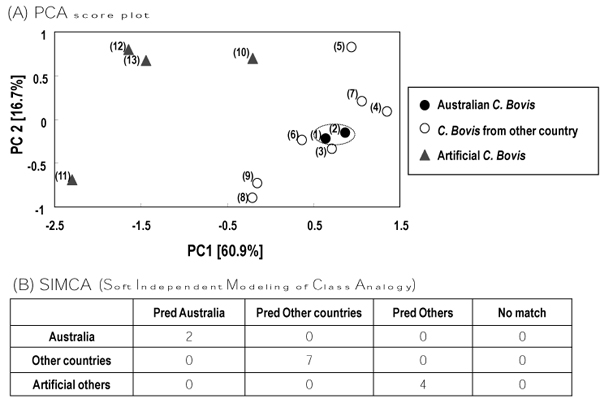
**Quality characterization of *Calculus Bovis* using pattern recognition techniques and multiple element data** (A) PCA score plot according to multiple element data. The number in parentheses matched to the number of samples in Table 1. Variables such as Na, Mg, Al, K, Ca, V, Cr, Mn, Fe, Co, Ni, Cu, Zn, Ge, As, Mo, Hg and Pb have been used as discriminating factors. Percentages in square brackets were contribution values. (B) Multivariate characterization by SIMCA. Symbols: Australian *C. Bovis*; (●); other countries (○); artificial samples (▲). Each point represented the mean values. (n = 3).

In total, 18 elements were determined in a range of 13 samples (9 natural *C. Bovis*, 4 artificial substitutes). The result indicated the variation among *C. Bovis* samples under different developmental conditions (Figure 2). PCA can classify 13 samples as shown in Figure 2-A. Thirteen samples were separated by their origin, Australian materials, other countries and substitutes. Australian materials were classified in the group of samples from other countries. The elements, Mn, Zn and Al, were found by PCA to contribute the classification of 13samples. A satisfactory classification using SIMCA was obtained both for Australia *C. Bovis* and the substitutes, namely 100 % of cases correctly classified (Figure 2-B).

### The role of taurine on the cardioactive efficacy of *Calucus Bovis*

In this study, we have used natural *C. Bovis* originated from Australia for ensuring its efficacy. The major constituents of this extract were bile acids (cholic acid: CA, deoxycholic acid: DCA, taurocholic acid:TCA) and taurine. The average bile acid concentration in Australian *C. Bovis* extract (0.1mg/ml ) was equivalent to 0.32 mM CA,0.32mM DCA and 0.24 mM TCA, respectively (not data shown).

**Figure 3:**
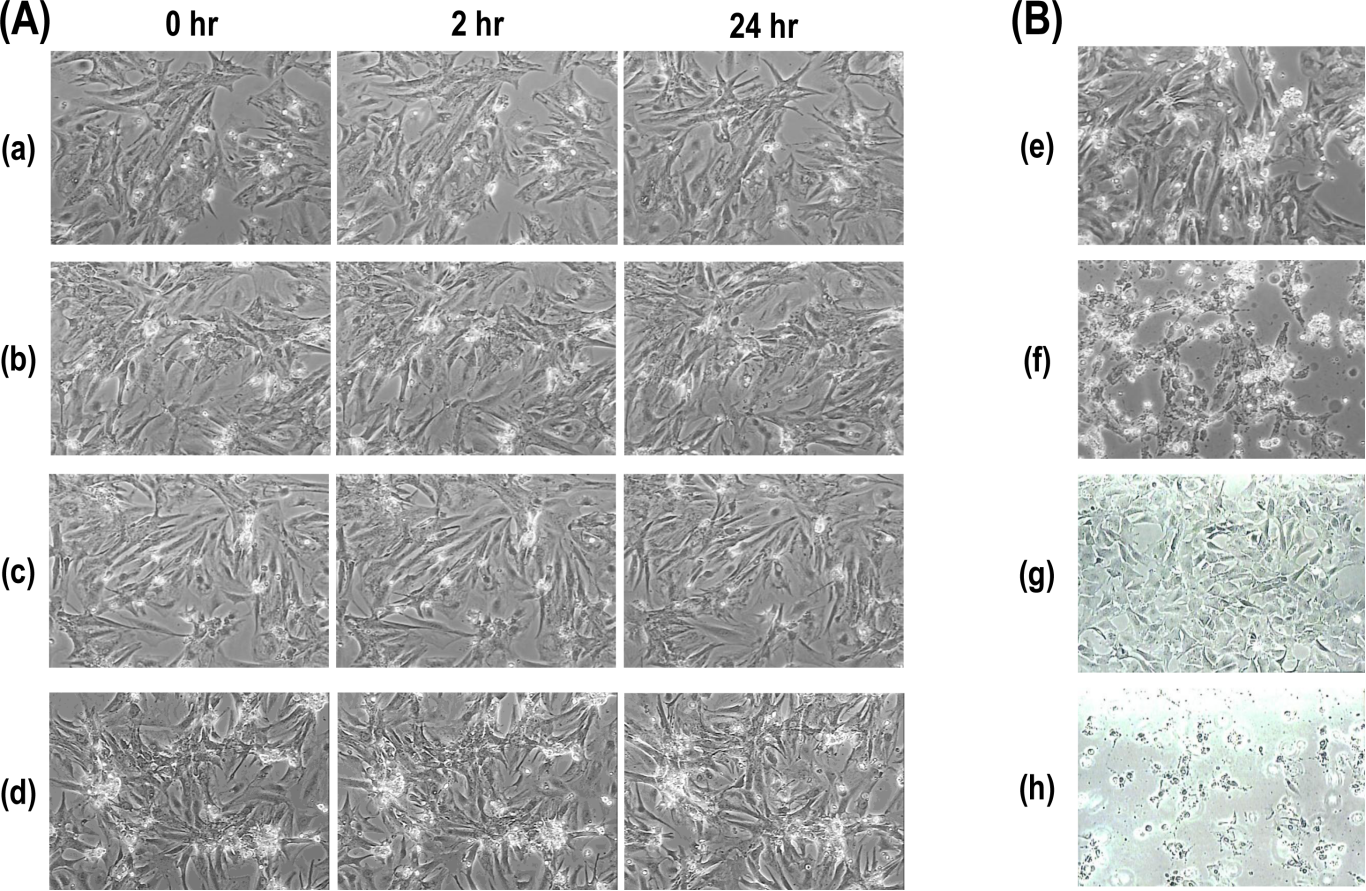
**Morphological changes of cardiac cells induced by Australian *Calculus Bovis* and deoxycholic acid** (A) Cardiomyocytes were incubated with increasing concentrations of Australian *C. Bovis*-extract: (a) 0 mg/ ml; (b) 1x10^-2^ mg/ml; (c) 5x10^-2^ mg/ml; (d) 1x10^-1^ mg/ml. The morphological changes of cardiomyocytes were recorded using phase contrast micrographs observed at a magnification of x100. (B) Cells were exposed to medium containing DCA (0.1 mM). Cardiac myocytes: (e) 0 h, (f) 1 h. Cardiac fibroblasts: (g) 0 h, (h) 1 h.

**Figure 4:**
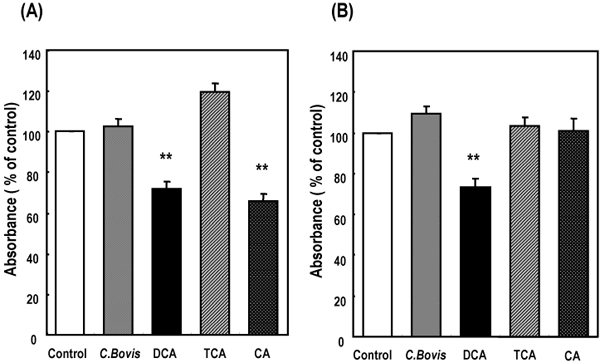
**Influences of *Calculus bovis* -related substances on viability of cardiac cells** Cells were incubated with *C. Bovis*-ext. and three bile acids for 48 h. Values were expressed as percent of control, in which the control cells were untreated. Data expressed means ±SEM obtained from of 12-14 samples from triplicate experiments. Cardiac fibroblasts (A) and myocytes (B) were treated with *C. Bovis* (1x10^-1^ mg/ml), DCA (0.1 mM), TCA (0.5 mM) and CA (0.5 mM), respectively. **P<0.01 compared with *C. Bovis*-ext.

To assess whether *C.Bovis* extract had cytotoxic effects on cardiac cells, morphological injury and cell viability using MTS assay were measured. Figure 3-A showed the influence of different doses of *C.Bovis* extract on the morphology of cultured cardiomyocytes. Addition of *C.Bovis* extract to the culture medium to give a final concentration of 0.01-0.1mg/ml had no change during 24 h (Figure 3-A).  At the dose of 0.1 mg/ml, treatment with *C.Bovis* extract for 48 h barely influenced the growth of cardiac myocytes and fibroblasts despite the bile acid contents of 0.2~0.3mM (Figure 4). The presence of 1mM DCA showed pronounced injury, including ballooning and cell lysis on cardiac myocytes and fibroblasts after 1 h (Figure 3-B).

Cardiac myocytes and fibroblasts treated for 48 h with CA or DCA caused cell injury, as reflected by changes in appearance and a reduction of viability detected by the MTS assay (Figure 4). The cell viability of both cells was reduced by approximately 70 % when the cells were treated with 0.1mM DCA, whereas TCA at a concentration of 0.5 mM had no effect over a 48-h period. Treatment with 0.5 mM CA caused a decrease in viability of fibroblasts by 66 % of control after 48 h. Cardiomyocyte was not significantly altered its viability by treatment with 0.5 mM CA for 48 h.

**Figure 5:**
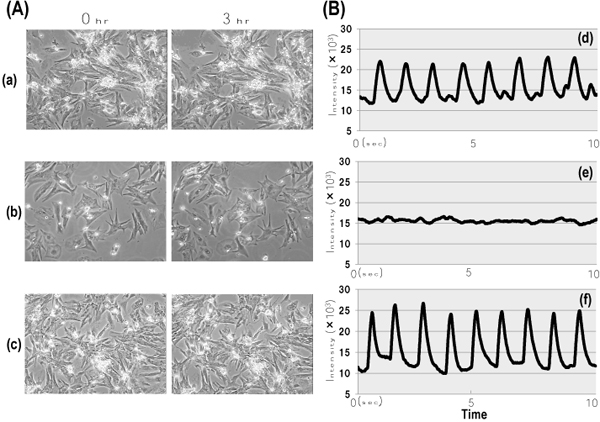
**Effects of cholate and *Calculus bovis* on beating of cardiac myocytes.  **Cardiomyocytes were exposed to medium containing CA or *C.Bovis*-extract for 3 h. The morphological changes (A) and beating status (B), untreated cells, (a, d); CA 0.5 mM, (b, e); *C.Bovis*-extract 1x10^-1^ mg/ml, (c, f)

Figure 5 shows a typical photograph and the beating status recorded by a photosensor equipped with microcomputer on cardiomyocytes. Changes in morphology and beating status were estimated for each cell on 12 points and expressed as percent of the total points of cells observed (not data sown). As shown Figure 5-B-d, untreated cells showed the rhythmic beating detected by vertical displacements. There is no significant inhibition on the beating status when 0.1 mg/ml of *C.Bovis* extract is administered (Figure 5-B-f). All cells in observed 12 points with/without *C.Bovis* kept to beat synchronously over a 3 h period without morphological changes. However, CA at a concentration of 0.5 mM caused a 50 % beat cessation of cardiomyocytes (Figure 5-B-e).

In China Pharmacopoeia, bile acids are the major active components and CA has been used as the chemical marker for quality control of *C. Bovis* and its substitutes [4]. Taurine is also provided as an ingredient of artificial *C. Bovis*.  In the present study, taurine content in several samples of natural *C. Bovis* and its substitutes varied widely between 0.035~104.7 mg/g. The mean taurine concentration for Australian natural *C. Bovis* in the study was 0.32 ±0.19 mg/g *C.Bovis* (2.56 μmol/g). The values for the highest and lowest taurine concentrations were 3.92 mg/g of Chinese and 0.035 mg/g of Mexican one, respectively.

**Figure 6:**
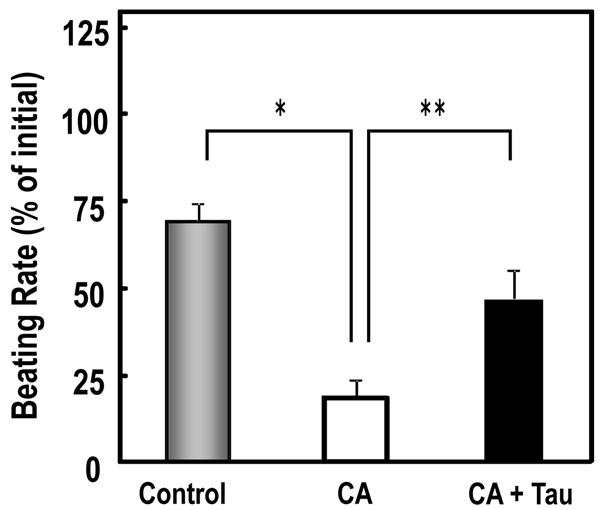
**Effect of taurine on the beat inhibition of cardiomyocytes induced by cholic acid** Cells were incubated for 0.5 h in medium containing CA (0.5 mM) and taurine (1 mM). Data were expressed as means ±SEM obtained from of 20 samples from triplicate experiments.  *P<0.01 compared with control, **P<0.01 compared with CA.

We have previously reported on the anti-arrhythmic actions of *C. Bovis* and suggested these effects are partly mediated by 0.1 mM-taurine [3]. Therefore, we tested the effects of taurine on CA-induced beating abnormality. In cardiomyocytes, 0.5 h exposure of CA markedly decreased the velocity ratio of beating from 69% to 18%, whereas the simultaneous addition of 1 mM taurine largely prevented the decrease, restoring the beating ratio to 47% (Figure 6).

## Discussion

Natural *C. bovis* is obtained as a valuable by-product from animals used for meat production. As the natural occurrence is very rare, great efforts have been taken for some substitutes of natural *C. Bovis*. In 2004, it was reported that 98 % of *C. Bovis* used in China was artificial [2]. However, due to the different developmental conditions, chemical constituents of substitutes might be different from those natural *C. Bovis*, which thus may lead to the variation of therapeutic effects.  In Japan, all materials of *C. Bovis* are imported from several countries such as Australia, Latin America, Africa, India and China [11].  Empirically, Japanese importers evaluated Australian *C. Bovis* as a high quality product without scientific reasons. The disordered use and abuse result in the loss of original pharmaceutical actions and therapeutic values of this natural product. Therefore, to ensure the quality of *C. Bovis* and its substitutes, an efficient quality control approach is urgently needed. Correctly characterizing the traditional animal materials is the inevitable starting point for studying *C. Bovis*.

First, we have characterized Natural *C. Bovis* originating from Australia using pattern recognition techniques and major and trace elements data (Figure 1, 2). PCA and multi-elemental focus was effective in discriminating *C. Bovis* samples derived from different habitats. There is general agreement among the experts/importers on crude drugs that Australian *C. Bovis* is quite a good quality on the basis of traditional knowledge. Interestingly, natural grouping of Australian samples was observed (Figure 2). Namely, the Australian ones had better batch uniformity than other habitats and were composed of fewer elements. The result indicated the variation among *C. Bovis* samples under different developmental conditions such as natural or artificial.

Secondly, we have used Australian* C. Bovis* for assessment on its bioactive compounds.  Chemical and pharmacological investigations on *C. Bovis* resulted in discovering several kinds of bioactive components, *i.e*. bile acids, bilirubin and some inorgamic salts [1,12,13]. CA has been used as the chemical marker for quality control of *C. Bovis* in the provisions of Chinese Pharmacopeia (Table 2) [4]. Taurine and CA are also one of the ingredients for artificial substitutes (Table 2) [4]. Since the content of CA,DCA and taurine in *C. Bovis* extract derived from different sources can vary enormously, its effect on cardiomyocytes needs to be analyzed after normalization based the value of taurine or CA/DCA. This is particularly important because CA/DCA and taurine have opposite effect on cardiomyocytes.

In the present study, cardiac cells treated with CA or DCA caused cell injury, as reflected by the morphological change and a reduction of cell viability (Figure 3, 4). Rat cardiac cells incubated with *C. Bovis* extract (0.01-0.1mg/ml) maintained a normal morphology, viability and beating pattern (Figure 3, 4, 5). However, cardiac cells treated with CA or DCA caused cell injury, as reflected by the morphological change and a reduction of cell viability. TCA, a major constituent in *C. Bovis*, also caused loss of synchronous beating, bradycardia and cessation of contraction in cultured rat cardiomyocytes [14,15]. We demonstrated a reduced rate of contraction and proportion of beating cells when rat cardiomyocytes were exposed to CA. 0.5 h exposure of CA markedly decreased the velocity ratio of beating, whereas the simultaneous addition of 1 mM taurine significantly prevented the decrease (Figure 6). Our previous data reported that *C. Bovis* extract was effective in protecting against the abnormal beating induced high Ca^2+^, and its efficacy was interfered by an inhibitor of taurine transport, bata-alanine [3,16]. It has been recognized that taurine may have some beneficial effects due to, for instance, its antioxidant or anti-apoptotic capacity [17-20]. It is plausible that the relationship between taurine and the bile acids contribute to the therapeutic effect of *C. Bovis*.

Finally, this work provided some references for the quality control of *C. Bovis* materials. For practical purposes and for conservation reasons [21], it is desirable to find acceptable substitutes to *C. Bovis* used in traditional medicines. And it is necessary to make use of modern scientific tools to establish verifiable synthetic substitutes as sustainable replacements for medicinal resources. Future work would be focused on the different pharmacological actions of these natural *C. Bovis* and the substitutes of some spurious breeds.

## Conclusions

The present work provided some references for the quality control and the efficacy of *C. Bovis*. PCA and multi-elemental focus was effective in discriminating *C. Bovis* samples derive from different habitats. Taurine partly attenuated the harmful actions of bile acids. It is plausible that the relationship between taurine and the bile acids contribute to the therapeutic effect of *C. Bovis*.

## Abbreviations

***C.Bovis***: *Calculus Bovis*; **ICP-MS**: inductively coupled plasma mass spectrometry; **PCA**: principal component analysis; **SIMCA**: soft independent modeling of class analogy; **CA**: cholic acid; **DCA**: deoxycholic acid; **TCA**: taurocholic acid; **GCA**: glycocholic acid; **TDCA**: taurodeoxycholic acid

## Competing interests

This work was supported in part by OTC Self-Medication Promotion Foundation 2009 and a grant from Tochimoto Tenkaido Co. (Osaka Japan).

## Authors’ contributions

K.T. and Y.A. conceived the experiments. K.T. and Y.A. performed the experiments with K.S. and analysed the data together with M.K. and T. S. S.W.S helped to edit the manuscript.
